# Engaging and supporting standardized patients involved in equity-seeking healthcare training: a qualitative study

**DOI:** 10.5116/ijme.67ab.596e

**Published:** 2025-03-20

**Authors:** Nicole Last, Urmi Sheth, Amy Keuhl, Aaron Geekie-Sousa, Derya Uzelli Yilmaz, Sandra Monteiro, Matt Sibbald

**Affiliations:** 1 Department of Medicine, Faculty of Health Sciences, McMaster University, Ontario, Canada; 2 Educational Services, Faculty of Health Sciences, McMaster University, Ontario, Canada; 3 Department of Fundamentals of Nursing, Faculty of Health Sciences, Izmar Katip Calebi University, Izmar, Turkey

**Keywords:** Psychological safety, standardized patients, human simulation, equity-seeking healthcare, diversity

## Abstract

**Objectives:**

This study aims to explore the perspectives of standardized patients previously involved in equity-seeking healthcare training simulation activities to better understand how stakeholders can engage and support standardized patients in the delivery of educational opportunities for healthcare professionals related to equity, diversity, and inclusivity.

**Methods:**

A qualitative research study was conducted utilizing semi-structured interviews with persons (N=15) who self-identified as being involved in the development and/or the delivery of simulations related to equity, diversity, and/or inclusion. Participants were recruited via email using internal and public lists for standardized patient programs and through snowball sampling. Interviews were recorded and transcribed verbatim and transcripts were analyzed qualitatively in an iterative coding process, anchored by direct content analysis methodology, and informed by three theoretical perspectives: the standardized patient journey, psychological safety, and empowerment theory.

**Results:**

We constructed three main themes: (1) safety should be prioritized throughout the journey; (2) empowerment is key to engagement; and (3) standardized patient trainers are central connectors for safety and empowerment.

**Conclusions:**

Through the perspectives of standardized patients, this study has provided insight on strategies to engage and support those participating in equity-seeking simulations. Focused attention on safety and empowerment is warranted, with trainers having a critical role in empowering standardized patients to succeed in equity-seeking simulations. Future research should continue to explore best practices surrounding engaging, supporting, and retaining individuals involved in equity-seeking healthcare training, including comprehensive training for trainers on how to ensure, maintain, and restore standardized patients’ psychological safety.

## Introduction

Standardized patient (SP) programs are increasingly being called upon by health professions training programs to support learning and assessment opportunities which integrate equity-deserving groups. As a result, there is an increased demand for SPs to participate in equity-seeking educational activities. Disparities across all areas of healthcare related to race, ethnicity, sex, gender, age, religious beliefs, mental health status, and other minoritized characteristics are a major concern, and healthcare training programs are trying to address this through curricular reconstruction.^1^^,^^2^ Teaching healthcare trainees how to engage with equity-deserving groups and understanding and valuing equity, diversity, and inclusivity (EDI) principles is essential for preparing these future healthcare professionals to provide patient and family-centered care to increasingly diverse populations.^3^ Simulation-based education allows learners to address EDI-related topics through its immersive nature, which enhances the transfer of learning to practice and offers rich opportunities for structured learning and debriefing.^4^ SP simulation as a pedagogy can promote EDI by allowing learners to practice engaging with sensitive topics in a safe learning environment; this is particularly important for equity-seeking healthcare training with SPs.

Previous studies have demonstrated the effectiveness of working with SPs to teach specific EDI-related skills.^5^^-^^7^ SP encounters can provide learners with valuable opportunities to practice providing equitable patient-centered care in a safe, judgement free environment, where learners are able to make mistakes and the risk of causing harm to real patients is reduced. However, as pointed out by Picketts and colleagues^8^ and congruent with previous work by Sibbald and colleagues.^9^ SPs who are recruited to engage in equity-seeking simulation-based training can actually be placed in vulnerable or uncomfortable situations; this merely shifts the potential for harm from ‘real’ patients to SPs.

Until recently, there has been little interest in the SP experience of participating in EDI-related simulations, and how SP programs can support them. A review conducted by Picketts and colleagues^8^ addressed diversity and inclusion in simulation and offered guidance related to ethical and psychological safety concerns when working with SPs from diverse and priority communities from a simulationist perspective. Another recent study, focused on quality enhancement, conducted focus groups with SPs to identify gaps in inclusivity, structural equity, and cultural humility, as perceived by SPs.^10^

However, a gap in SP methodology literature still exists relating to how to engage SPs in equity-seeking healthcare training and how to best support them through the training and simulation process. The present study was designed to provide insights and recommendations to consider when engaging SPs in equity-seeking healthcare training and how SP programs can support them throughout the journey. Providing insight into SPs’ experience to encourage the integration of equity-seeking SP simulation-based education will allow us to develop some guidance to promote a just and inclusive culture of learning for all involved.

## Methods

### Study design

We conducted a theory informed, exploratory study using semi-structured interviews of key informants around how to support SPs recruited for equity-seeking healthcare training simulations (refer to Appendix for the interview guide). The analytic process was guided by direct content analysis in an iterative design to establish key themes.

#### Recruitment strategy

Recruitment focused on SPs who had been involved in delivering simulations related to EDI. As long as the individual self-identified as being involved in an EDI-related SP activity, they were offered an interview, whether or not they were explicitly recruited for the activity based on their identity, appearance, and/or lived experience. We recruited using internal and published lists for national and international SP programs. Guided by our study aims, sampling specificity, and analysis strategy, we anticipated requiring 15-20 interviews to reach saturation. Written informed consent was obtained from all participants. The study was approved by the Hamilton Integrated Ethics Review Board (#12614).

### Data collection

The interview guide (Appendix) was developed by the research team based on a review of the literature^2^ and prior qualitative work with educators.^9^ The interview guide was piloted and revised prior to data collection. Semi-structured interviews were conducted by two authors (NL and US). Interviews were audio recorded using the Zoom platform, transcribed verbatim, and anonymized. Participants were asked about their background related to SP work, specific EDI-related SP activities they have been involved in, their experiences relating to SP recruitment, training, delivery, and debrief processes, and perspectives around supporting SP’s psychological safety. The analytic team met with the interview team monthly to review 2-4 transcripts, evolve the interview guide, inform sampling, and determine when saturation was reached.

#### Ethical considerations

All participants were assigned an identification number, and personal information was not associated with participants’ interview data. After interviews were transcribed and verified, audio files were deleted and all transcripts were cleaned of any identifying information. All collected data was stored in a secure and confidential location. Participants were offered a $50 gift card for their time. In addition, prior to starting each interview, participants were reminded that they could stop or pause the interview at any time.

### Data analysis

Three theoretical perspectives informed the analysis: the SP journey, psychological safety, and empowerment theory.

(1)      The SP journey – In this study, we use the SP journey as a framework that describes the many internal stages and processes involved in employing SPs to participate in simulation-based education, from the perspective of an SP program and identified best practices. We identified six distinct stages: onboarding and orientation, recruitment and scheduling, role-specific training, portrayal (or delivery), learner feedback, and debriefing. Onboarding and orientation refers to the process of recruiting and hiring SPs, and providing basic skills training related to SP simulation. Recruitment and scheduling SPs for roles generally occurs after an SP has been hired and oriented and involves finding the ‘best fit’ for the role, based on the scenario and role requirements. Once scheduled, SPs typically undergo role-specific training, either in a group session or one-on-one with an SP trainer, to discuss scenario details, including case content and specific role-portrayal instructions (e.g., appearance, affect), and dry run the scenario (i.e., role-play). The goal of role-specific training is to ensure the SP is adequately prepared to execute the portrayal (or delivery) of the scenario with learners at a predetermined time and location. Post-portrayal, SPs are often expected to provide learner feedback based on their experience as the patient, sometimes noting specific examples of things that were said and/or done during the portrayal. The process of debriefing refers to stepping out of the role and allowing for reflection on how the simulation made the SP feel; this process may occur with learners through a facilitated discussion, internal to the SP (self-debriefing), and/or with assistance from an SP trainer or other member of SP program staff. Using the framework of the SP journey allowed us to identify many different ways to support SPs participating in equity-seeking training activities for healthcare professionals.

(2)      Psychological safety – Psychological safety, in the context of establishing a safe space in which learners can make mistakes without fear of consequences, is essential for optimal learning within simulation and is well described in the literature.^11^^,^^12^ Less prevalent, however, are discussions around psychological safety and potential threats to safety in the context of SPs participating in EDI-related simulations. Though EDI-related simulations involving SPs can be invaluable in helping learners better understand and provide care to individuals from equity-deserving groups, such simulations are not void of risk or unintended effects on SPs.^13^^,^^14^ Psychological risk has been defined as “a perceived or actual feeling of mental threat as a result of participation in a simulation which can mean feeling unsafe”;^15^ it can include feelings of shame or humiliation,^16^ may result from threats to dignity or acts of discrimination, and can put individuals at risk of retraumatization.^8^ During the interviews, the participants provided their thoughts on how to ensure and maintain psychological safety throughout the SP journey when participating in EDI-related simulations. In the discussion, we will contextualize participants’ perspectives pertaining to psychological safety based on the level of interaction (individual, team, or organizational), as described by Kolbe and colleagues.^12^

(3)      Empowerment theory – As a general framework, empowerment includes processes and structures that enhance participation and improve goal achievement.^17^ We used empowerment theory to help distinguish between empowering processes and empowered outcomes at the individual, organizational, and community levels. We also attempt to understand and categorize the consequences of disempowering experiences described by SPs. In addition, we give special focus to empowerment at the individual level of analysis: psychological empowerment. As a construct of empowerment, psychological empowerment includes community change, capacity building, collectivity, and learning about controlling agents and acting to influence those agents; this requires active engagement in one's community and an understanding of one's sociopolitical environment.^18^ Empowering processes allow people to freely create or receive opportunities to influence the decisions that affect their lives and control their destiny.^17^ We used psychological empowerment as a model to: (1) understand the agency and voice of SPs who have participated in EDI-related simulations, (2) understand SP’s experiences of empowerment and disempowerment, and (3) determine strategies to support SPs involved in equity-seeking healthcare training.

We employed a staged approach to analyzing the data, moving from qualitative description to direct content analysis. The initial stages involved open coding to identify key themes using shared online documents and an initial coding scheme representative of the three theoretical perspectives as a guide. Monthly research team meetings facilitated discussion around codes and linkages, which enabled the subsequent transfer of the group’s coding into qualitative analysis software (HyperRESEARCH 4.5.2) by two authors (NL, US). Both NL and US discussed their coding findings upon the completion of each transcript to identify any coding disputes. Disputes were discussed as a group to achieve a collective consensus. Research team meetings were recorded to allow the group to reference prior discussions and ensure the diversity of perspectives from the analytic team was being represented in the final coding. Code tables and mind-mapping of all transcripts were developed (US, NL) and reviewed by the analytic team. The team discussed patterns and emerging themes suitable to organize and represent the data. We continued this iterative process until saturation was achieved, defined as (1) no new themes or ideas emerging from the last few interviews, and (2) themes could be constructed that were well supported by the transcripts and meaningful for SPs, educators, and SP programs.

#### Rigour and Trustworthiness

The research team itself was made up of diverse perspectives from both inside and outside of marginalized groups and both within and outside of healthcare education. NL has a master’s degree within rehabilitation science and practical experience as a SP Trainer. At the time of data collection, US was an undergraduate student with previous interviewing experience. AK works as a project officer and has experience facilitating research projects. AGS has practical experience as a SP trainer. DUY, SM, and MS are healthcare educators and DUY has training in nursing; SM and MS have training in medicine.

All team members were invited to share their individual insights on each transcript. Reflexivity was promoted through the critical and constant reflection of potential biases, through journaling and monthly group discussions where the research team openly discussed personal judgements and belief systems and how they might influence interpretation of the data. We acknowledge that researcher subjectivity has influence on each step of the research process and cannot be erased; our aim was to minimize researcher biases and their influence on the outcome of this study by having open and honest discussions about our backgrounds and personal beliefs.

## Results

We constructed three themes based on 15 interviews with participants from Canada and the United States (Table 1): (1) safety throughout the journey (2) empowerment is key to engagement; and (3) SP trainers central to safety and empowerment.

(1)  Safety throughout the journey 

Participants described how both physical and psychological safety can be fostered during EDI-related simulations through transparency, autonomy, competence, boundaries, and connection. Participants’ perspectives of these proponents of safety will be applied to the relative stages of the SP journey (i.e., onboarding and orientation, recruitment and scheduling, role-specific training, delivery, learner feedback, and debriefing).

Onboarding and orientation – In the early stages of the SP journey, SPs valued the opportunity to opt out of recruitment calls for certain simulation activities or topics:

“But this is a case where I would think in the onboarding to ask not only are there certain medical conditions or do you have certain scars that might preclude you from participating in certain kinds of events, but are there areas that you would rather we don't call upon you for…. Being able to announce it confidentially then so you're not always revisiting it with that person might be helpful.” [PAR 5]

Additionally, part of ensuring SP safety in the early stages include explicitly informing (and reminding) SPs of their rights. Participant 5 recalls their SP orientation experience,

“And I do remember that, at one point, he was very specific and very adamant about if you feel uncomfortable or any of those situations, like our rights as SPs.”

Because faculty/instructors have the potential to threaten SP safety, one participant suggested orientation for them as well,

“I wish that the instructors would also participate in some sort of orientation so that everyone's on the same page in terms of how you interact with SPs.” [PAR 1]

**Table 1:** Participant characteristics (n=15)

Characteristics		Participants
Geographic distribution		
Canada		
	Alberta	5
	Manitoba	3
	Ontario	1
	Saskatchewan	1
United States		
	Connecticut	5
Identifies with marginalized group		
Yes		7
	Gay, queer, and/or genderqueer	2
	Disabled	1
	Living below the poverty line	1
	Neurodivergent	1
	Woman	1
	Black	1
	East Indian	1
	Inca Indian	1
	Latino	1
	Puerto Rican	1
	Southeast Asian	1
No		5
Missing data		3
Education level		
	Diploma	1
	Bachelors	1
	Masters	2
	Doctorate	1
	None	1
	Missing data	9
Years in SP work		
	0-5	8
	6-10	3
	11-15	1
	Missing data	3
Has had formal training related to EDI		
	Yes	6
	No	5
	Missing data	4

Recruitment and scheduling – Participants highlighted the importance of being provided sufficient details to give informed consent and also emphasized SPs themselves have a responsibility to know their individual capabilities, both physically and psychologically:

“I think being open to different scenarios is really important as well, but also understanding where your hard-lined boundaries are. Sometimes you don't know until you get asked, so just taking the time instead of just leaping forward and being like, "Yes!" just to understand where potentially triggers might happen for you.” [PAR 8]

During recruitment for a specific role, screening and excluding individuals from participating because it could be triggering for them based on past experiences (e.g., this case deals with the topic of abortion so you should not participate in this activity if you have previously had an abortion) was viewed as paternalistic and bad practice: “But that also just feels like a bit patronizing to be like, ‘I'm gonna make this decision for you,’ rather than respecting their own self-determination” [PAR 1]. Instead, the need to empower SPs through transparency of what a role involves, a safe space to make that informed decision for themselves, was emphasized.

Training and delivery – Clear boundaries related to role-portrayal maintain safety for SPs and are perceived to support learner safety as well. Some SPs described uncertainty about knowing ‘how far is too far,’ and establishing those boundaries during training was necessary to ensuring SPs felt prepared for and competent in their role-portrayal. Group dry runs were seen as a helpful strategy to ‘work out all the kinks and ask questions’ [PAR 15]. Frequent reminders of SPs’ rights and check ins from SP trainers had a big impact on SPs’ emotional well-being. Participant 6 commented on the impact of SP trainer check ins, “And not in the sense of, you know, policing us, but, ‘Just wanna check in. Are you okay with this?’ Just that little bit makes you feel very supported at all times” [PAR 6].

Learner feedback – When transitioning from delivery to learner feedback, participants recognized the importance of removing themselves from the role, while still providing feedback from the role’s perspective, "This patient felt this way. This patient would have felt like this.” [PAR 9] The role faculty has in fostering safety was also highlighted, noting that faculty can promote safety by being prepared to have open discussions with learners about issues relating to equity-deserving groups: “When they give feedback to the learners, they should have some insight, if a learner asked a question in more of an offensive way…. So I think faculty facilitators, and all that, should also be familiar with these particular issues [PAR 15].

Debriefing – Post session, or debriefing, was recognized as a crucial time for maintaining safety by providing opportunities to resolve triggers, engage in sensemaking, and bring closure and move forward without resentment or other negative emotions. Some participants felt that just having an outlet to provide feedback to the SP trainer after a simulation was enough for them, as long as a culture of trust and support was previously established, “Just being able to give that feedback, even if it is informal. Just having at least a culture of an environment where you feel you can do that I think is enough. It doesn't have to be more formalized than that” [PAR 6].

Main findings from this theme included participants’ desire for transparency about what a role entails, the potential risks, and the learning objectives of the simulation; this information was considered important to make an informed decision around participation in the simulation. Additionally, clear boundaries related to role-portrayal were critical to ensure SPs felt prepared and maintaining safety for SPs and perceived to be important for learners’ safety as well. Access to someone familiar with the role, whether an SP who portrayed the role previously, or someone with lived experience, and to a community of SPs were suggested strategies to ensure SPs were adequately prepared for role-portrayal. Participants also identified time outs, role separation, structuring of the session, and removing oneself from the role when transitioning from delivery into learner feedback as key components of safety during simulation.

(2)  Empowerment is key to engagement

Participants involved in EDI-related simulations, such as in the form of playing the role of a person involved in sex work, a person who is transgender, or a person of a marginalized racial or ethnic group, had different motivations for portraying these roles. Commonly, participants saw these roles as an opportunity not only to help healthcare trainees, but also the larger community of people who commonly experience healthcare disparities:

“What I hope is that they open the eyes of the students to individuals that they may not have had contact with or experience with during their life experience …. give them the opportunity to show more empathy, and then to really give them language and skills and knowledge that their life experience probably did not have.” [PAR 9]

Acknowledgement of the value of SPs and their important role in training healthcare professionals was one identified approach to engaging SPs in EDI-related simulation. Speaking of their onboarding experience, one participant stated:

“It was extraordinary because not only did they talk about what obviously the job responsibilities would be, but they really talked about the role of an SP in the medical education of the [US University] students and then on a larger national scale, what that meant for the next generation of physicians and improvements in medical pedagogy. And so you felt very much valued as an SP right from the get-go, so that was great.” [PAR 6]

Participants who identified as a member of a marginalized group appreciated when they were recruited for a role with which they could identify; however, those feelings diminished if the SP’s voice was not heard or when their input was ignored, particularly when providing feedback on scenario content:

“It's a bit disheartening to see the case again a couple of years later exactly the same…. I feel like sometimes it feels like we have the conversations about it and it feels like that's enough, and then no actual change happens.” [PAR 3]

Many participants called for educators and programs to listen to the SP experience and be open to their suggestions:

“Listen to your SPs because we're the ones that are so deep into the scenario, and we're the ones that can identify the gaps…. just [be] open to it no matter how uncomfortable it is, at least hear somebody out, 'cause maybe they'll have some good ideas that you want to implement.” [PAR 8]

SPs could be empowered to continue to engage in equity-seeking healthcare training activities through continuously consulting them about their experiences by asking questions and actually making an effort to implement necessary changes. For instance, one participant stated, “I appreciate someone asking, ‘what could we do differently?’ or ‘were you comfortable in that situation?’” [PAR 9] and another suggested SPs could be asked, “’Do you have any additional notes with these scenarios that will even better help the next SP who comes in and takes on this role?’ I think taking our opinions and suggestions on that would be very helpful.” [PAR 8]

Receiving reassurance and feedback regarding role portrayal seems to help SPs feel confident and more likely to continue to engage in activities,

“And also I think approaching each individual SP and just making them aware that if there are perhaps any gaps in their knowledge or something they're uncomfortable with, that all of the questions that they have should be answered.” [PAR 8]

(3)  SP trainers were central to safety and empowerment 

Participants’ level of connectedness and relationship with the SP trainer impacted whether an SP felt safe and empowered throughout the SP journey.

The SP’s relationship with the trainer was central to safety:

“Yeah, and if the relationship between SP trainers and SPs is a positive one… I think that can make someone feel more comfortable if they felt like it was necessary to talk to someone.” [PAR 1]

SPs appreciated check ins throughout the SP journey, as a strategy to ensure the SPs comfort and safety are being maintained:

“As we were starting to do it, I realized I can't do this, I'm going to fall apart…. I called [the SP trainer] and I said, ‘I am so sorry, but I can't do this.’ And so I think that's the piece that the trainers need to really say, even if we say yes, and just keep checking in with people.” [PAR 5]

Another participant highlighted the accessibility of SP trainers for support: “Or maybe an open line to your trainer that you really do feel like you can just pop a question saying, ‘Wow, I'm really holding onto this interaction I had today. I just wanna talk about it with somebody’” [PAR 11]. This same participant noted value in SP trainers taking initiative to pre-schedule a debrief session:

“Not just, you know, ‘You can debrief,’ but, ‘We are going to debrief after this sensitive role.’ So that was wonderful. That was a great experience when I had that. Because I did not even know that I needed to unpack some of the emotional stuff going on. So that is something that I think should happen more often than not.” [PAR 11]

SP trainers themselves needed to be resourced to provide empowerment and safety: “I sure wish the trainers could have more resources at their fingertips to be able to say, ‘Gee, I would like to talk to somebody in this organization about this,’ and I don't think that they do all the time” [PAR 11]. In addition, training for SP trainers was thought important:

“I think that all trainers should have some education or diversity training because... I don't know how you can train someone for a role if you don't understand some of these groups.” [PAR 15]

“I think if you are to have some of the SP organizers have that training to debrief on psychological safety would go a long way because, realistically, these programs aren't gonna hire outside mental health therapists, people that they don't have to pay money to, it just doesn't make financial sense for them. So what would make the most sense is incorporating some sort of module or training to the people running the program, so that if you do encounter this, you have someone with knowledge on how to de-escalate the situation or make it so that the SP is feeling more psychologically supported.” [PAR 14]

SP trainers were seen as responsible for incorporating experts into the process to ensure the role is an accurate and safe representation:

“And also, yeah, I guess partnering with people with lived experience to then write cases that aren't just the run-of-the-mill situations. 'Cause not every trainer is gonna have experience with... even if there was the most diverse SP trainer, they don't have experience with everything.” [PAR 1]

## Discussion

**Figure 1:**
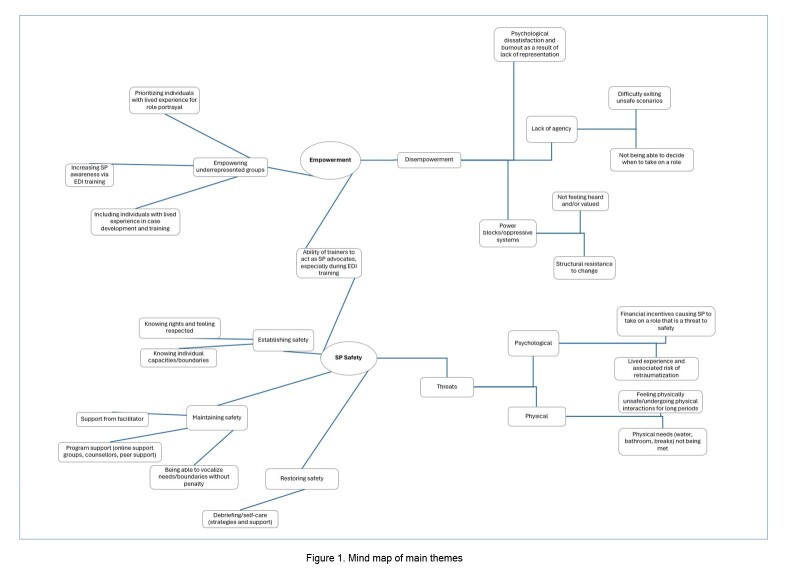
Mind map of main themes

Healthcare training programs have a duty to prepare trainees to meet the needs of a diverse population.^1^ SP simulation is commonly used for meeting these mandates and teaching communication and clinical skills related to EDI in healthcare.^2^^,^^4^ As such, SPs play an invaluable role as proxies for real patients in health professions education; this can put SPs in a vulnerable position, especially if they are being recruited for specific roles because of their appearance, lived experience, or identity. In the present study, we interviewed 15 SPs who self-identified as participating in the development and/or delivery of EDI-related SP activities with a goal of exploring ways SP programs can effectively engage and support SPs involved in these sorts of educational activities. Findings from these interviews indicated that engaging and supporting SPs in simulations of equity-deserving groups requires consideration of SP safety, SP empowerment, and the central roles of the SP trainers in empowering SPs and establishing, maintaining, and restoring SP safety (Figure 1).

The concept that simulation needs to be carried out in a safe environment is not new. However, previous conversations in the literature regarding a safe learning environment and psychological safety in simulation activities have focused on learners and the perception that learners can express themselves without fear of negative consequences, that it is safe for learners to take risks, and that learners will not be embarrassed, judged, rejected, or otherwise punished for not knowing or asking questions.^12^^,^^19^^-^^21^ Safety in simulation does not only apply to learners, but also to the vulnerable work that SPs engage in to create educational opportunities. In this study, we identified several instances throughout the SP journey, from recruitment to deroling, where SP safety was described as being present, threatened, and requiring restoration. For example, SPs may feel an obligation to accept a role that has the potential to threaten their psychological safety because of financial need and/or fear of letting others down. In these instances, the importance of informed consent about what a role entails, permission to ask for time outs, and the assurance of safe spaces for SPs to step out of the session were highlighted. Similar perceptions were highlighted in a previous study on the perspectives of SP trainers and faculty involved in the development of EDI-related SP simulation activities.^9^ Consideration is required for how safety is not only created but also how it is maintained and restored when threatened. Kolbe and colleagues^12^ discuss implicit and explicit strategies that contribute to psychological safety before, during, and after debriefing as part of simulation-based training. Findings from the present study build on Kolbe’s model for debriefing and open the discussion on how to manage threats to psychological safety beyond debriefing (e.g., in training and during the simulation scenario), specifically in the context of SPs involved in equity-seeking work.

SPs recruited for roles portraying equity-deserving groups are volunteering to make themselves vulnerable and risk retraumatization and exposure to unintended oppression, particularly if the SP identifies with the role they are being asked to portray. Finding strategies to prevent and counteract these forces through empowerment is vital to the wellbeing of SPs and the success of SP programs in recruiting and retaining SPs and delivering high-quality and diverse educational services. A recent review of the role of SP programs in promoting EDI noted the recruitment, training, and processes for ensuring safety differ for SPs who portray versus those who share (i.e., have lived experience) and that SPs being asked to portray a role with which they identify should be intrinsically motivated in a way that aligns with curricular learning objectives and that these SPs should be provided supports that facilitate sharing in a safe way for all involved.^2^ Similarly, empowering SPs involved in EDI-related simulation requires a social contract with the educational system that not only gives voice in the co-creation of cases^9^ but also a process that involves actively seeking out feedback from SPs, listening to their experiences, and adapting simulation content accordingly. Not doing so was seen as disempowering and demotivating, and a threat to relationships with and retention of SPs from traditionally marginalized and underrepresented groups. In a study on the perspectives of transgender and genderqueer SPs, Noonan and colleagues^14^ describe a mutually beneficial relationship between the medical education system and SPs from gender minorities wherein SPs described hope, empowerment, and engagement as positive aspects of participation. However, it is imperative that the entire education team is prepared to work with diverse SP groups, including using inclusive language, and that the expectation is not for SPs from traditionally marginalized and underrepresented groups to teach team members.^14^ In the present study, strategies to empower SPs included opportunities for institutional learning, such as EDI training for all stakeholders, including SP program staff, learners, and faculty members.

While the ‘how to’ of engaging and supporting SPs participating in equity-seeking healthcare training is complex and requires supports from and collaboration of many key stakeholders, the role of SP trainers was seen as critical to SP safety and empowerment. Based on SP trainer’s expectations and implicit responsibilities, we have coined the title “SP learning environment navigators” to reflect the expanded role that these team members play in the system. To fulfill these duties, “SP learning environment navigators” need access to adequate training supports and institutional support and oversight. More research on intended and actual roles and responsibilities of SP trainers and how institutions can best support these team members so they can adequately engage and support SPs involved in equity-seeking healthcare training is required.

### Limitations

This study is not without limitations. First, due to the methods of recruitment, this study does not capture the perspectives of SPs who could engage in equity-seeking work but choose not to nor does it include the perspectives of those who have been disenfranchised by this work and are no longer part of an SP program. Second, the data reflects the lived experience of SPs with varying intersectionality, diversity training, educational experience, access to supports, and other diversifying characteristics. The voices represented here do not and cannot speak for those who may experience other barriers to safety, empowerment, and participation beyond those identified in this study. Lastly, all participants in the present study lived in North America at the time, and therefore the results of this work may not be generalizable to SP programs and SPs in other contexts. However, advocacy for increased support for SPs involved in equity-seeking healthcare training can be universally applied.

### Implications for future research

This work provides insight for SP programs and educators surrounding SP experiences participating in equity-seeking healthcare training simulation and includes potential strategies for engaging and supporting SPs throughout their participation in these activities. Building on the current study, more studies are needed to continue to capture diverse perspectives of participating in equity-seeking healthcare training simulations, especially those from traditionally underrepresented and marginalized groups. Studies that explore the variances and unique requirements related to engagement and support throughout the SP journey of different equity-deserving groups are particularly important.

## Conclusions

The conversation around managing psychological safety in educational settings has historically focused on learners. SPs are an integral part of medical education worldwide and are increasingly being asked to participate in simulations integrating equity-seeking training into the curriculum. SPs engaged in equity-seeking simulations warrant focused attention on safety and empowerment throughout the SP process, from recruitment to deroling. SP trainers are in an opportune position to foster relationships with and be allies to community members from marginalized and underrepresented groups but require access to adequate training and resourcing for an expanded role as SP learning environment navigators. We are hopeful this research will spark discussion and provide a way forward in terms of engaging, and supporting, and retaining SPs involved in equity-seeking healthcare training.

### Acknowledgements

The authors are extremely grateful to and would like to thank the SPs who participated in this study. We would also like to thank Farhan Bhanji (Faculty of Medicine and Health Sciences, McGill University) and Faran Khalid (Department of Medicine, McMaster University) for their contributions to this research. This work was supported by a strategic initiatives grant from the Royal College of Physicians and Surgeons of Canada (20-SIG-03).

### Conflict of Interest

The authors declare that there is no conflict of interest.

## Supplementary material

Supplementary file 1 Appendix. Interview Guide (Standardized Patients)
