# Supplementary material for: Engaging and supporting standardized patients involved in equity-seeking healthcare training: a qualitative study
Source: Int J Med Educ. 2025 Mar 20;16:64–74. doi: 10.5116/ijme.67ab.596e (PMC12700524; doi:10.5116/ijme.67ab.596e)
Supplement: Supplementary file 1 — Appendix. Interview Guide (Standardized Patients) [file ijme-16-64-S1.pdf]

Appendix

Interview Guide (Standardized Patients)

Preamble and Informed Consent Process

Thank you for agreeing to participate in this interview today. I appreciate you taking the time.

You should have already received a copy of the informed consent form. Have you had a chance to read it? Do you have any questions that you would like to ask before you consent to participate? When you are ready, could you please sign the form and email me back a copy?

I anticipate this interview will take approximately 60-90 minutes of your time. I want to assure you that what you share in our interview will be treated as confidential. You will be assigned an ID number so that your name is not associated with your interview.

There are no right or wrong ways to respond. Please answer as fully as possible. If you would prefer not to answer a question, just say "pass". I can pause or stop the interview at any time, upon your request.

Is it okay if I start the audio-recording now? I may also take some written notes while we're talking.

Background related to SP work and EDI

To start, I would like to understand a bit more about your background and work related to standardized patients and equity, diversity, and inclusivity.

- How long have you been a standardized patient and how did you initially become involved in working as a standardized patient?
- What did the hiring and intake process look like for you? How did you feel about the process?
- How do you think SP programs can help with EDI issues in healthcare training programs?

Enrollment

- What sort of equity, diversity, and/or inclusivity standardized patient activities have you been involved in?
  - What did the engagement or enrollment process look like?
  - Why did you decide to take on these sorts of roles? How did taking on these sorts of roles make you feel? Did you have any hesitations?
  - What was the experience of training for these roles like for you?
    - What are the goals of SP training, in your opinion?
    - How could the SP training and feedback process have been improved for you?
    - What training or lived experiences do you draw on in doing this work?
- "Nothing about us without us" is often quoted when doing EDI work. What are your thoughts on this in terms of portraying a role representing a group that you may not personally identify with?

Psychological safety

- If an SP is playing a role with which they have lived experience with, what are your thoughts around ensuring psychological safety and any negative emotions that might arise?
  - Has this happened to you, and if so, how were you supported through it?

Learner feedback

- What kind of feedback do you provide to learners for a role in which you have lived experience? What about a role in which you do not have lived experience?

We are coming close to the end of the interview. Given your experiences as an SP, we are interested in your impressions of how best to work with SPs for healthcare training:

- What do you think about SPs as a vehicle for EDI training? What is the best way to use SPs for EDI objectives? Are there situations where SPs should not be used?
  What advice would you give curricular leads or faculty interested in using SPs for EDI training?
  Any advice for SP program managers or trainers? Any advice for other SPs?

Other Potential Prompt Questions (to be used throughout the interview)

	• Can you tell me more about that?<br>• How did that make you feel?<br>• How did you know that was what you needed to do in that moment?<br>• What happened because of that?<br>• Was there anything about the experience that surprised you?<br>• Was there anything about that experience you wish you could change?<br>• How would that experience be different if that had not happened?<br>• Why do you think that is? What made you come to that conclusion?
**Conclusion**	
	• Those are all the questions I have for you right now. Did we miss anything? What questions do you have for me?
	I will be taking this recording and creating a transcript. I may contact you for clarification if there is a part of the recording I can't understand, or I need your help to clarify. Once I am finished the entire study, I will share the results.<br><br>Thank you again for your time.
